# The MSPDBL2 Codon 591 Polymorphism Is Associated with Lumefantrine *In Vitro* Drug Responses in Plasmodium falciparum Isolates from Kilifi, Kenya

**DOI:** 10.1128/AAC.03522-14

**Published:** 2015-02-11

**Authors:** Lynette Isabella Ochola-Oyier, John Okombo, Leah Mwai, Steven M. Kiara, Lewa Pole, Kevin K. A. Tetteh, Alexis Nzila, Kevin Marsh

**Affiliations:** a Kenya Medical Research Institute/Wellcome Trust Collaborative Research Program, Kilifi, Kenya; b Department of Immunology & Infection, Faculty of Infectious & Tropical Diseases, London School of Hygiene & Tropical Medicine, London, United Kingdom; c King Fahd University of Petroleum and Minerals, Department of Biology, Dhahran, Saudi Arabia

## Abstract

The mechanisms of drug resistance development in the Plasmodium falciparum parasite to lumefantrine (LUM), commonly used in combination with artemisinin, are still unclear. We assessed the polymorphisms of *Pfmspdbl2* for associations with LUM activity in a Kenyan population. MSPDBL2 codon 591S was associated with reduced susceptibility to LUM (*P* = 0.04). The high frequency of *Pfmspdbl2* codon 591S in Kenya may be driven by the widespread use of lumefantrine in artemisinin combination therapy (Coartem).

## TEXT

Chemotherapy is central to the treatment and control of Plasmodium falciparum malaria but faces the parasite's intrinsic ability to quickly develop resistance to antimalarials. The combination of artemisinin and lumefantrine (LUM) (Coartem) is the treatment of choice for uncomplicated malaria in much of Africa (1). However, parasites showing reduced LUM susceptibility have been reported in some countries (2–6). This has been associated with the selection of wild-type parasites at the P. falciparum chloroquine resistance transporter (*crt*) 76 locus (7, 8) and with at least a 2-fold increase in the frequency of the multidrug resistance 1 (MDR1) 86N mutants following treatment (2, 4, 5, 9). Recently, a single nucleotide polymorphism (SNP) in merozoite surface protein Duffy binding-like 2 (MSPDBL2) codon 591 (C591S) was shown to be associated with increased resistance to halofantrine, mefloquine, and LUM (10). Since LUM is now widely used in the treatment of malaria, it is important to understand the mechanisms of resistance to this drug. We therefore investigated the role of *Pfmspdbl2* in the response to LUM *in vitro* using Kenyan isolates and chloroquine (CQ) as a reference drug. *Pfmspdbl2* is a member of the MSP3 multigene family, including *mspdbl1*, *msp3*, and *msp6* (11). The associated proteins are expressed simultaneously (11) and potentially interact with other proteins on the merozoite parasite membrane in the invasion of the erythrocyte (12, 13). Thus, all 4 genes were included in our investigation.

Parasite genomic DNA was extracted using a QIAmp DNA blood minikit (Qiagen, United Kingdom). We amplified the full-length *Pfmspdbl1*, *Pfmspdbl2*, *Pfmsp3*, and *Pfmsp6* from 65 *in vitro* culture-adapted isolates with chemosensitivity data for CQ and LUM (7, 14) using the primers and PCR cycling conditions described in Table S1 in the supplemental material. PCR products were sequenced using BigDye Terminator v3.1 chemistry (Applied Biosystems, United Kingdom), and the resultant sequences were assembled and edited using SeqMan and aligned using MegAlign (Lasergene 7; DNASTAR, Madison, WI).

*Pfmsp3*, *Pfmsp6*, *Pfmspdbl1*, and *Pfmspdbl2* alleles were defined on the basis of their haplotype structure and associations with *in vitro* drug responses assessed using the Kruskal-Wallis test and the Bonferroni correction for multiple comparisons (15). Thus, *P* values of <0.001 remained significant. We also determined the median 50% inhibitory concentrations (IC_50_s) and the 95% confidence intervals (CIs) for each SNP that showed a significant association (*P* < 0.05) with the drugs tested. Only SNPs with a >5% minor allele frequency were included, and all analyses were conducted using Stata v.11 (StataCorp, College Station, TX).

**FIG 1:**
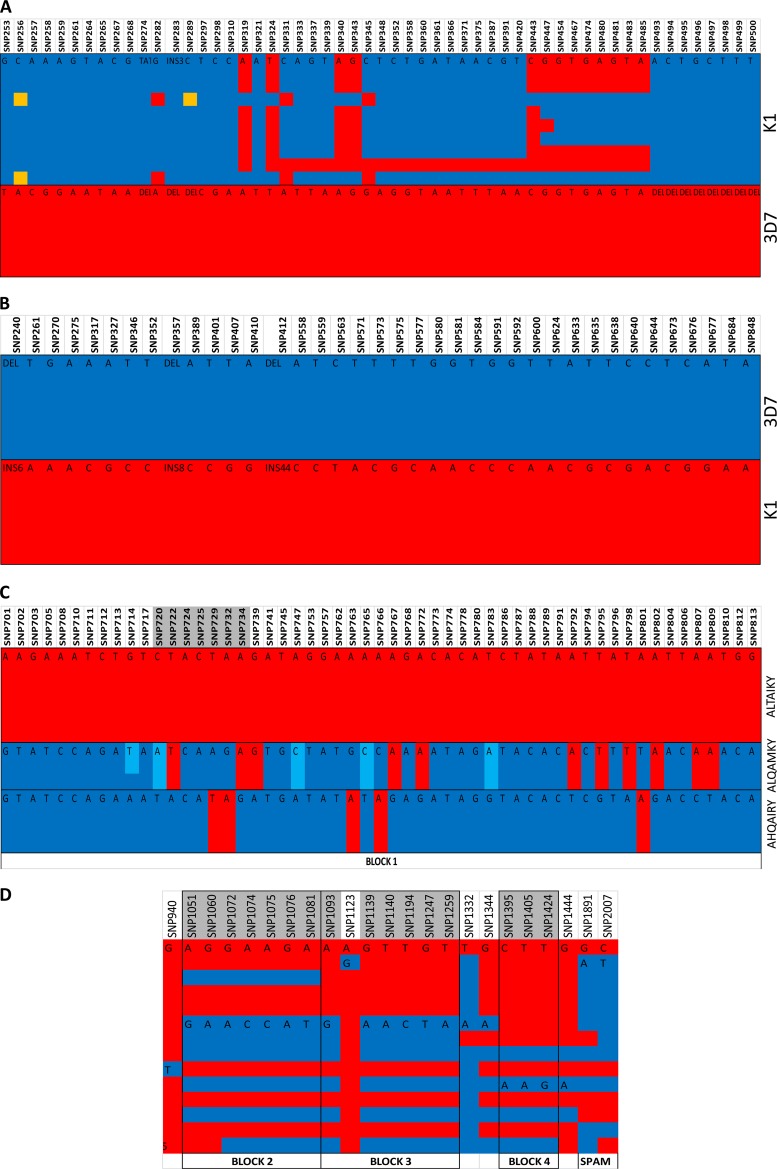

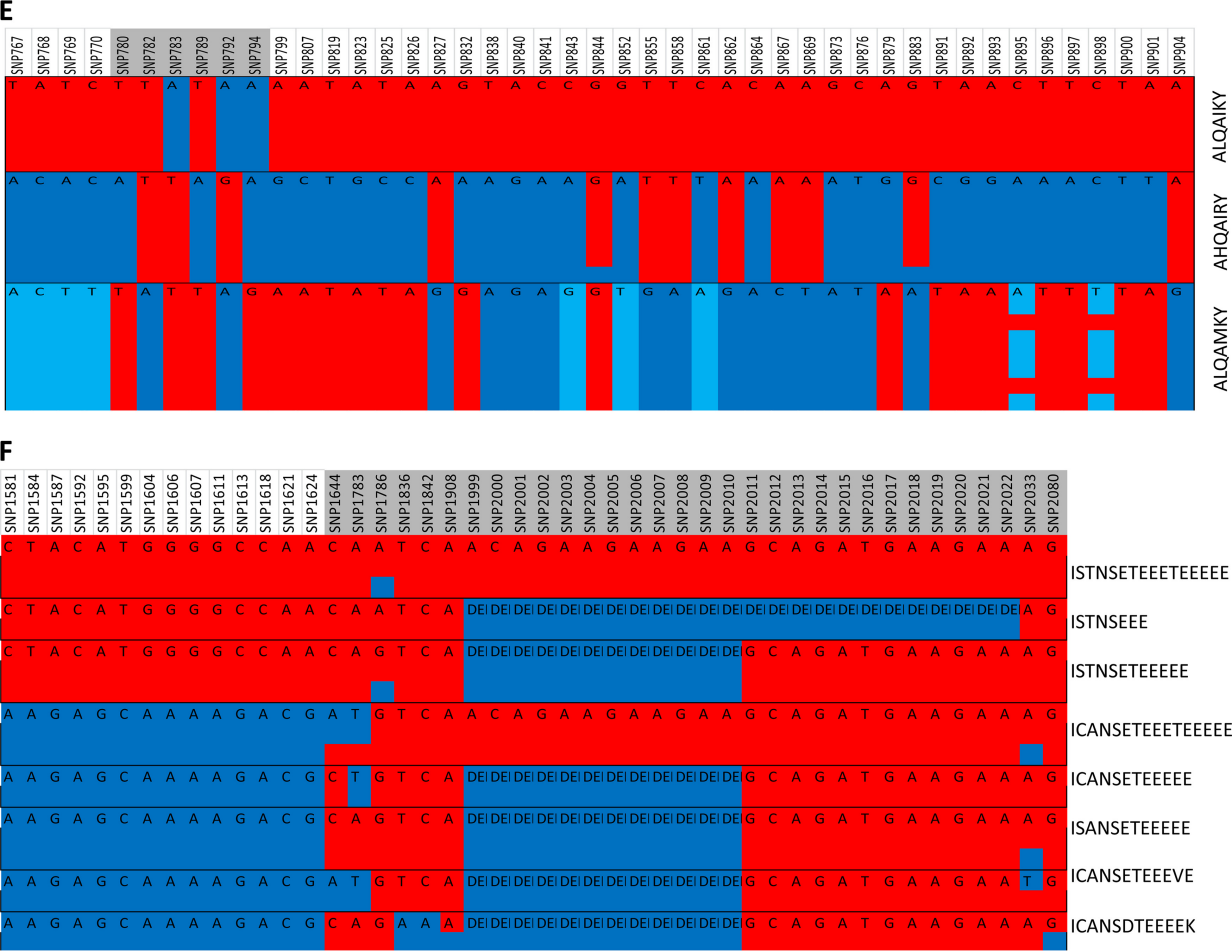
Linkage disequilibrium in the *Pfmsp3* multigene family. The haplotypes were generated from an analysis of sequenced SNPs. Haplotypes shown are *Pfmsp3* K1 and 3D7 (A), *Pfmsp6* K1 and 3D7 (B), *Pfmspdbl1* DBL domain AHQAIRY, ALTAIKY, and ALQAMKY (C), *Pfmspdbl1* 3′ DBL domain NEVRI, DKIQF, and NEIQF block 2, NGGRI and DEGIK block 3, TSV and TTG block 4, and the SPAM domain KN and EN (D), *Pfmspdbl2* DBL domain AHQAIRY, ALQAIKY, and ALQAMKY (E), and *Pfmspdbl2* SPAM domain 8 (F). Each column represents an SNP, each color in the column represents a different nucleotide, and each row represents an isolate sequence. The black outline depicts the allelic blocks. The columns shaded in gray are in linkage disequilibrium, and the amino acids from these polymorphisms were used to define the haplotypes. INS, number of nucleotides inserted (e.g., INS3 means 3 nucleotides); DEL, absence of sequence (i.e., a deletion).

All *msp3* and *msp6* SNPs analyzed were in linkage disequilibrium, representing two previously defined alleles, K1 and 3D7 (Fig. 1A and B, respectively) (16, 17), therefore precluding individual SNP analysis. The activity of all the drugs tested did not differ in parasites harboring 3D7 or K1 alleles in the *msp3* and *msp6* genes (Table 1). The SNPs of *Pfmspdbl1* and *Pfmspdbl2* were used to determine the actual loci within the haplotypes associated with changes in drug responses.

**TABLE 1:** Results of the drug sensitivity assays for chloroquine and lumefantrine compared to the *Pfmsp3*, *Pfmsp6*, *Pfmspdbl1*, and *Pfmspdbl2* haplotypes

Gene	Haplotype^*c*^	Chloroquine			Lumefantrine		
		*n* (%)	Median IC_50_ (95% CI)	*P* value	*n* (%)	Median IC_50_ (95% CI)	*P* value
MSP3	K1	14 (64)	36.27 (14.59–98.06)		14 (64)	67.34 (47.26–143.97)	
	**3D7**	8 (36)	23.29 (7.59–183.16)	0.78	8 (36)	82.7 (45.09–260.0)	0.68
MSP6	K1	11 (31)	80.05 (25.46–181.35)		11 (31)	67.56 (40.04–236.42)	
	**3D7**	25 (69)	54.94 (18.22–87.09)	0.21	25 (69)	97.85 (66.25–167.88)	0.36
MSPDBL1^*a*^							
BLOCK 1	AHQAIRY	6 (20.7)	83.56 (32.5–108.58)		6 (20.7)	74.55 (32.81–339.98)	
	**ALTAIKY**	17 (58.6)	31.62 (14.84–109.39)		17 (58.6)	124.32 (67.13–274.75)	
	ALQAMKY	6 (20.7)	56.32 (2.28–107.37)	0.55	6 (20.7)	197.62 (39.11–357.11)	0.43
BLOCK 2	NEVRI	20 (56)	85.7 (58.35–104.22)		20 (56)	75.07 (48.39–102.84)	
	**DKIQF**	13 (36)	14.99 (13.28–67.4)		13 (36)	169.29 (69.42–340.89)	
	NEIQF	3 (8)	109.46 (54.94–298.1)	0.0093^*b*^	3 (8)	75.1 (67.56–104.41)	0.06
BLOCK 3	NGGRI	23 (64)	85.23 (55.94–99.74)		23 (64)	84.60 (53.17–102.04)	
	**DEGIK**	13 (36)	16.77 (13.28–102.07)	0.068	13 (36)	104.41 (62.7–340.89)	0.054^*b*^
BLOCK 4	**TSV**	31 (84)	80.14 (30.46–91.55)		31 (84)	97.64 (68.23–140.13)	
	TTG	6 (16)	70.54 (10.53–279.59)	0.77	6 (16)	85.98 (52.36–402.73)	0.21
SPAM domain	**KN**	21 (64)	30.7 (15.3–89.1)		21 (64)	97.6 (68.4–115.3)	
	EN	12 (36)	103.3 (59.2–114.9)	0.007^*b*^	12 (36)	61.3 (36.9–270.6)	0.28
MSPDBL2							
DBL domain	**AHQAIRY**	10 (34.4)	81.55 (30.42–112.89)		10 (34.4)	72.07 (37.26–229.12)	
	ALQAIKY	11 (31.3)	54.94 (11.08–86.81)		11 (31.3)	104.16 (72.53–252.08)	
	ALQAMKY	11 (31.3)	41.58 (12.74–92.17)	0.25	11 (31.3)	50.19 (37.91–142.39)	0.17
SPAM domain	ISTNSETEEETEEEEE	2 (6)	8.41 (2.28–14.55)		2 (6)	248.05 (124.32–371.77)	
	ISTNSEEE	2 (6)	203.78 (109.46–298.1)		2 (6)	85.98 (67.56–104.41)	
	ISTNSETEEEEE	8 (29)	67.91 (16.16–98.52)		8 (29)	91.12 (66.47–238.47)	
	ICANSETEEETEEEEE	3 (9)	86.17 (83.06–104.5)		3 (9)	28.47 (22.95–36.76)	
	**ICANSETEEEEE**	3 (9)	92.96 (12.79–215.59)		3 (9)	48.59 (48.37–97.85)	
	ISANSETEEEEE	6 (19)	43.13 (11.51–109.58)		6 (19)	92.1 (59.18–227.23)	
	ICANSETEEEVE	5 (16)	30.73 (14.31–167.55)		5 (16)	59.54 (38.3–318.93)	
	ICANSDTEEEEK	2 (6)	15.8 (14.84–16.77)	0.14	2 (6)	294.57 (242.75–346.39)	0.034^*b*^

a Blocks 1, 2, and 3 are within the MSPDBL1 DBL domain, while block 4 is between the DBL and SPAM domains.

b Significant result (*P* ≤ 0.05).

c Boldface represents the 3D7 reference sequence.

*Pfmspdbl1* contained a single DBL domain (11, 13), defined by 3 haplotype blocks (Fig. 1C and D), a secreted polymorphic antigen associated with merozoites (SPAM) domain (codons 631 and 669), and a haplotype block between the DBL and SPAM domains (Fig. 1D). *Pfmspdbl2* also contained a single DBL domain (11, 13) and a SPAM domain (13), and we obtained sequence data from codons 127 to 520 (Fig. 1E) and 527 to 694 (Fig. 1F), respectively.

**FIG 2:**
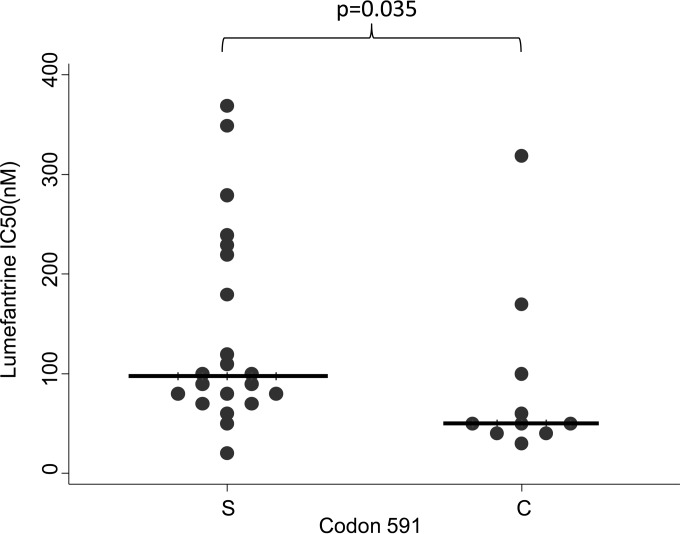
*Pfmspdbl2* codon 591 (*n* = 46) S allele is associated with reduced susceptibility to LUM (*P* = 0.035). The horizontal lines indicate the median drug IC_50_s.

The MSPDBL1 haplotypes (*n* = 36) showed an association with CQ (*P* < 0.01) and LUM (*P* = 0.05) drug activity, while the haplotypes of MSPDBL2 (*n* = 31) showed an association with LUM (*P* = 0.03) (Table 1). Twelve *Pfmspdbl1* SNPs (Table 2; see also Table S2 in the supplemental material) and 4 SNPs of *Pfmspdbl2* (Table 3; see also Table S3 in the supplemental material) were associated with both CQ and LUM. Notably, *Pfmspdbl2* SNP1783 (*n* = 31) codes for codon 591 (since the SNP followed 3 indels, 12 bp long), of which parasites containing serine were associated with reduced susceptibility to LUM (IC_50_, 97.6 nM; 95% CI, 77.7 to 199.8 nM; *P* = 0.04) (Fig. 2; Table 3). Codon 591S was also found at a high frequency (68%) in our population, similar to findings in Senegal (80% frequency) (10). This association of *Pfmspdbl2* codon 591S with reduced susceptibility to LUM in a different African population adds support to the findings of the study in Senegal and suggests that codon 591 may be a marker for the surveillance of LUM resistance. Importantly, though, codon 591 is not likely to be the causal variant conferring resistance to LUM. Van Tyne et al. (10) demonstrated that stable integrants containing PfMSPDBL2 C591 were more sensitive to mefloquine, halofantrine, and LUM than those with the 591S parasite. The extensive use of LUM in Africa may be the major driving force favoring the high frequency of the 591S mutation.

**TABLE 2:** *Pfmspdbl1* SNPs significantly associated with drug responses to chloroquine and lumefantrine

Codon(s)	SNP(s)	Nucleotide(s) (amino acid[s])^*a*^	Chloroquine				Lumefantrine			
			*n* (%)	Median IC_50_ (nM)	95% CI	*P* value	*n* (%)	Median IC_50_ (nM)	95% CI	*P* value
351, 354	1051, 1060	**G, A (DK)**	12 (32)	14.7	12.6–31.5		12 (32)	206	52.4–344.9	
		A, G (NE)	25 (68)	86.2	58.1–104.3	0.0013^*b*^	25 (68)	80.8	66.3–97.8	0.04^*b*^
358, 359	1072, 1074, 1075, 1076	**A, C, C, A (IQ)**	14 (38)	23.8	14.0–93.8		14 (38)	136.8	66.2–334.8	
		G, A, A, G (VR)	23 (62)	85.2	55.9–99.7	0.05^*b*^	23 (62)	84.6	53.2–102.0	0.04^*b*^
361	1081	**T (F)**	15 (41)	30.7	14.4–95.7		15 (41)	111.6	68.9–330.0	
		A (I)	22 (59)	85.7	56.4–102.3	0.08	22 (59)	82.7	48.5–98.2	0.03^*b*^
365	1093	**G (D)**	13 (36)	16.8	13.3–102.1		13 (36)	104.4	62.7–340.9	
		A (N)	23 (64)	85.2	55.9–99.7	0.07	23 (64)	84.6	53.2–102.0	0.05^*b*^
380	1139	**A (E)**	14 (37)	24.2	14.0–98.9		14 (37)	108	66.2–334.8	
		G (G)	24 (63)	84.1	48.2–96.1	0.12	24 (63)	87.7	60.2–110.5	0.06
380, 398, 416	1140, 1194, 1247	**A, C, T (EGI)**	14 (38)	24.2	14.0–98.9		14 (38)	108	66.2–334.8	
		T, T, G (GGR)	23 (62)	85.2	55.9–99.7	0.11	23 (62)	84.6	53.2–102.0	0.045^*b*^
420	1259	**A (K)**	15 (39)	31.6	14.4–95.7		15 (39.5)	111.6	68.9–330.0	
		T (I)	23 (61)	85.2	55.9–99.7	0.09	23 (60.5)	84.6	53.2–102.0	0.035^*b*^
444	1332	**A (K)**	26 (74)	84.1	31.1–99.7		26 (74)	104.3	77.7–206.6	
		T (N)	9 (26)	56.4	12.8–103.6	0.5	9 (26)	48.6	36.9–93.8	0.03^*b*^
631	1891	**A (K)**	24 (67)	30.5	14.9–82.5		24 (67)	100.7	76.9–160.3	
		G (E)	12 (33)	103.3	59.2–114.9	0.0025^*b*^	12 (33)	61.8	36.9–270.6	0.17
669	2007	**T (N)**	19 (58)	30.7	15.5–82.3		19 (58)	97.6	73.3–138.1	
		C (N)	14 (42)	106.4	78.6–123.8	0.0015^*b*^	14 (42)	58.1	38.0–240.1	0.19

a The bold letters highlight the 3D7 reference alleles.

b Significant result (*P* ≤ 0.05).

**TABLE 3:** *Pfmspdbl2* SNPs significantly associated with drug responses to chloroquine and lumefantrine

Codon(s)	SNP	Nucleotide(s)^*a*^ (amino acid[s])	Chloroquine				Lumefantrine			
			*n* (%)	Median IC_50_ (nM)	95% CI	*P* value	*n* (%)	Median IC_50_ (nM)	95% CI	*P* value
591	1783	**T (C)**	10 (32)	58.9	14.4–147.1		10 (32)	50.1	37.3–146.1	
		A (S)	21 (68)	55.8	16.2–90.6	0.77	21 (68)	97.6	77.7–199.8	0.035^*b*^
608, 610	1836, 1842	**TC (NS)**	28 (93)	68.2	30.1–95.7		28 (93)	87.7	61.6–109.6	
		AA (KR)	2 (7)	15.8	14.8–16.8	0.23	2 (7)	294.6	242.8–346.4	0.046^*b*^
667	2011–2010	**ACAGAAGAAGAA (TEEE)**	29 (94)	54.9	16.4–84.0		2 (6)	86	67.6–104.4	
		DEL	2 (6)	203.8	109.5–298.1	0.04^*b*^	29 (94)	90.8	59.1–138.1	0.94

a The bold letters highlight the 3D7 reference alleles.

b Significant result (*P* ≤ 0.05).

The observed inverse drug relationship of 4 *Pfmspdbl1* codons (Table 2) associated with resistance to CQ, for instance, codons 351 and 354 (NE, IC_50_, 86.2 nM; 95% CI 58.1 to 104.3 nM; *P* = 0.001), and susceptibility to LUM (NE, IC_50_ = 80.8 nM, 95% CI, 66.3 to 97.8 nM; *P* = 0.04) is reminiscent of the previously described inverse relationship of wild-type CQ parasites showing resistance to LUM (8, 18, 19). This inverse relationship between drugs shown by SNPs of *Pfmspdbl1* most likely occurs on a backdrop of the underlying inverse relationship driven by CQ and LUM, since they are drugs that have been widely used for malaria treatment. Consequently, LUM-artemisinin may select for wild-type *crt*, associated with CQ sensitivity (20) and implicated as a marker of LUM tolerance (2, 7), suggesting that LUM is likely to confer resistance via a different mechanism.

Not surprisingly, none of the *Pfmsp3*, *Pfmsp6*, *Pfmspdbl1*, or *Pfmspdbl2* SNPs were in linkage disequilibrium with the K76T *crt* locus (data not shown). Additionally, the MSP3 gene family contains multiple high-frequency polymorphisms, which would increase the probability of random associations with drug activity. Furthermore, since *Pfmspdbl2* has shown evidence of being under balancing selection and is likely to be under immune pressure (21), its role in immunity cannot be ignored. However, it remains to be determined if the C591S mutation can be used as a surveillance marker of LUM resistance in the field.

## Supplementary Material

Supplemental material
