# Supplementary material for: The MSPDBL2 Codon 591 Polymorphism Is Associated with Lumefantrine In Vitro Drug Responses in Plasmodium falciparum Isolates from Kilifi, Kenya
Source: Antimicrob Agents Chemother. 2015 Feb 11;59(3):1770–5. doi: 10.1128/AAC.03522-14 (PMC4325780; doi:10.1128/AAC.03522-14)
Supplement: Supplemental material [file supp_59_3_1770__index.html]

Supplemental material 

# The MSPDBL2 Codon 591 Polymorphism Is Associated with Lumefantrine *In Vitro* Drug Responses in Plasmodium falciparum Isolates from Kilifi, Kenya

## Supplemental material

**Files in this Data Supplement:**

- Supplemental file 1 -

  List of primers and PCR conditions used in amplification of *Pfmsp3* multigene family target sequences (Table S1).

  XLSX, 17K
- Supplemental file 2 -

  Associations between *Pfmspdbl1* SNPs and chloroquine and lumefantrine (Table S2).

  XLSX, 22K
- Supplemental file 3 -

  Associations between *Pfmspdbl2* SNPs and chloroquine and lumefantrine (Table S3).

  XLSX, 23K
